# Human Papillomavirus Type 16 Early Protein E7 Activates Autophagy through Inhibition of Dual-Specificity Phosphatase 5

**DOI:** 10.1155/2022/1863098

**Published:** 2022-03-10

**Authors:** Chunting Hua, Qiaoli Zheng, Jiang Zhu, Siji Chen, Yinjing Song, Stijn van der Veen, Hao Cheng

**Affiliations:** ^1^Department of Dermatology, Sir Run Run Shaw Hospital, School of Medicine, Zhejiang University, Hangzhou, China; ^2^Department of Microbiology, Collaborative Innovation Center for Diagnosis and Treatment of Infectious Diseases, School of Medicine, Zhejiang University, Hangzhou, China

## Abstract

Consistent high-risk human papillomavirus (HPV) infection leads to various malignant cancers. Autophagy can promote cancer progression by helping cancer cells survive under stress or induce oncogenic effects when mutations or abnormalities occur. Mitogen activated protein kinases (MAPKs) can transduce various external or intrinsic stimuli into cellular responses, including autophagy, and dual-specificity phosphates (DUSPs) contribute to the direct regulation of MAPK activities. Previously, we showed that expression of DUSP5 was repressed in HPV16 E7-expressing normal human epidermal keratinocytes (NHEKs). Here we show that clinical HPV16 E7-positive precancerous and cancerous tissues also demonstrate low DUSP5 levels compared with control tissues, indicating that the inverse correlation between HPV16 E7 and DUSP5 is clinically relevant. We furthermore investigated the autophagy response in both DUSP5-deficient and HPV16 E7-expressing NHEKs. Confocal microscopy and Western analysis showed induction of LC3-II levels, autophagosome formation and autophagy fluxes in DUSP5-deficient NHEKs. Furthermore, Western analysis demonstrated specific induction of phosphorylated ERK in DUSP5-deficient and HPV16 E7-expressing NHEKs, indicating that HPV16 E7-mediated repression of DUSP5 results in induced MAPK/ERK signaling. Finally, phosphorylated mTOR and ULK (S757) were reduced in DUSP5-deficient NHEKs, while phosphorylated ULK (S555) and AMPK were increased, thereby inducing canonical autophagy through the mTOR and AMPK pathways. In conclusion, our results demonstrate that HPV16 E7 expression reduces DUSP5 levels, which in turn results in active MAPK/ERK signaling and induction of canonical autophagy through mTOR and MAPK regulation. Given its demonstrated inverse correlation with clinical cancerous tissues, DUSP5 may serve as a potential therapeutic target for cervical cancer.

## 1. Introduction

Human papillomavirus (HPV) is a double-stranded DNA virus with a circular genome that contains three functional regions: the early transcription region, the late transcription region and the upstream regulation region. Based on the general outcome of HPV infections, HPV is divided into two groups: high-risk HPV (HR-HPV) and low-risk HPV (LR-HPV). HR-HPV is associated with malignancies such as oropharyngeal cancer and cervical cancer, and its genome frequently integrates into the host-cell chromosome during malignant progression. Although bivalent, quadrivalent and nonavalent vaccines preventing HPV-induced cervical cancer are becoming more widely available, there are currently still no therapeutics. Therefore, a better understanding of HPV biology is still essential. Some of the abnormally expressed proteins associated with HPV infection may serve as potential prognostic markers or therapeutic targets. The early transcription region encodes the early genes E1, E2, E4, E5, E6 and E7, which are essential for replication, transcription and transformation. Furthermore, oncogene E7 can also regulate other cellular proteins or interfere in their normal function [1], thereby impacting cellular proliferation and autophagic responses.

Autophagy, a dynamic cellular recycling system that accounts for removal, degradation, and recycling by lysosomal activity, plays a crucial role in cellular homeostasis at basal levels and adaptation to stress conditions such as aging and pathogen infections. Increased numbers of autophagic vesicles and LC3-positive puncta in virus infected cells indicates activation of autophagy and could be interpreted as viral targeting and degradation [2–5]. Also, the role of autophagy in enhanced innate recognition of viral pathogens [6], negative inflammatory regulation to avoid excess inflammation [7], and enhanced antigen presentation [8, 9] further clarify various virus-autophagy interactions.

Many viruses have evolved measures to suppress or exploit autophagy for the benefit of infection [10, 11]. Also, autophagy is a key driver of malignant transformation in virus-related tumor development, which is selectively triggered during viral replication to increase the ability to live in a high-energy-required environment [12–14]. For instance, previous studies showed that classical swine fever virus [15] and bovine ephemeral fever virus [16] induce autophagy during viral replication, while inhibition of virus-induced autophagy with aspirin suppressed viral replication. Similarly, HPV also exploits the autophagic machinery for infection and proliferation of infected epithelial cells, although the role of autophagy appears to be less clear-cut as it can act as both tumor suppressor and a promoter [17]. Furthermore, the relationship between HPV16 E7 and autophagy also seems contradictory. Expression of HPV16 E7 in normal human keratinocytes resulted in stronger LC3 staining and an increased incidence of LC3 puncta, which is evidence for induced autophagy-related fluxes [18]. Furthermore, HPV16 E6/E7 played a positive role in autophagy activity in cervical cancer cells via accelerating autophagosome formation and degradation [19]. In contrast, depletion of HPV16 E7 from the W12 and CaSki cervical carcinogenesis models induced conversion of LC3B-I to LC3B-II and reduced the levels of p62, which similarly showed activation of autophagy fluxes [20]. HPV E6/E7-positive keratinocytes generate strong replicative and oxidative stresses, which are counteracted by autophagy activity [21]. Therefore, expression of oncogenes may promote aberrant cell proliferation and trigger metabolic stresses that increase energy requirements, which need to be tightly balanced by autophagy-related processes.

In our previous study, we showed that dual-specificity phosphatase 5 (DUSP5) is downregulated in HPV16 E7-expressing human keratinocytes [22]. DUSPs belong to a heterogeneous family of protein tyrosine phosphatases, which serve as major modulators of the mitogen activated protein kinase (MAPK) signaling pathway through specific dephosporylation of various MAPKs, such as extracellular signal regulated kinase (ERK), c-Jun N-terminal kinase (JNK) and p38 [23, 24]. MAPKs are serine/threonine-specific kinases that have evolved to communicate environmental and developmental signals from the membrane into adaptive and programmed responses in the nucleus [25, 26]. Different members of the DUSP family show distinct substrate specificities, tissue distribution and subcellular localization, and different modes of inducibility by extracellular stimuli [27].

Autophagy, which serves as the quick responsor to metabolic stress and nutrient status, is regulated by various kinases, such as mammalian target of rapamycin complex 1 (mTORC1) and MAPK. Several studies have shown that inhibition of DUSP1 activates autophagy [28–30], and it seems therefore that regulation of the expression or activity of DUSPs can be a promising alternative strategy to modulate autophagy. For instance, it was shown that during a *Mycobacterium bovis* Bacillus Calmette-Guerin (BCG) infection, DUSP5 inhibited the formation of autophagosomes by suppressing phosphorylation of signaling molecules in the ERK1/2 signaling cascade [31].

DUSP5, which is expressed at very low levels in cervical cancer cells, has been verified to be negatively modulated by lncRNA ARAP1 antisense RNA 1 and to contribute to cell proliferation and migration [32]. In our current study, we consistently found a negative correlation between DUSP5 and HPV16 E7 expression levels and DUSP5 deficiency resulted in induced autophagy fluxes. Modulation of DUSP5 may therefore serve as a novel strategy for autophagy regulation.

## 2. Materials and Methods

### 2.1. Clinical Samples of Patients and Ethical Statements

This study has been approved by the ethics committee of the Sir Run Run Shaw Hospital (Approval no. 20210330-39) and is in accordance with the Declaration of Helsinki. Briefly, samples were randomly picked after clinical diagnosis of cervicitis, cervical intraepithelial neoplasia or cervical cancer. Paraffin-embedded specimens of HPV16-negative or -positive cervical tissues were classified after DNA extraction and nested PCR verification for HPV detection.

### 2.2. DNA Extraction and Nested PCR

DNA from formalin-fixed and paraffin-embedded (FFPE) patient tissues was extracted using the FFPE Tissue Kit (QIAamp, Germany) following manufacturer's instructions. Briefly, tissue sections were collected in sterile tubes and xylene was used to remove paraffin. Tissues were subsequently lysed under denaturing conditions with proteinase K and incubated at 90°C to reverse formalin crosslinking. Nested PCR was performed using primer pairs MY09 (CGTCCMARRGGAWACTGATCa)/MY11 (GCMCAGGGWCATAAYAATGGa) and GP5 + (TTTGTTACTGTGGTAGATACTAC)/GP6 + (GAAAAATAAACTGTAAATCATATTC) for the first and second PCR reactions, respectively. PCR reactions were performed in a 25 *μ*L volume using Taq enzyme (CWBIO, China). The reaction mixture was first heated to 95°C for 5 min, followed by 35 cycles of 30s denaturation at 95°C, 30s annealing at 57°C and 45 s extension at 72°C, with a final extension step at 72°C for 10 min. The second reaction was identical, except annealing was performed at 58°C. PCR products were run on a 2% agarose gel for visualization (Supplementary Figure 1).

### 2.3. Immunohistochemistry Staining

Paraffin-embedded tissue slides (3 *μ*m thick) were heated at 65°C for 30 min and deparaffinized in xylene. After rehydration in a graded series of ethanol solutions, slides were immersed in 0.01 M sodium citrate buffer (pH 6.0) inside a pressure-cooker for 15 min to achieve heat-induced antigen retrieval. Tissues were then incubated with 3% hydrogen peroxide for 30 min, rinsed with PBS, and incubated with 5% nonimmune goat serum for 30 min inside a moist chamber. Tissues were subsequently incubated overnight with anti-DUSP5 (ab200708, 1 : 500; Abcam) or anti-HPV16 E7 (1 : 500) at 4°C. Finally, the tissues were washed and incubated with secondary anti-rabbit IgG (Gene Tech, China) for 30 min, followed by staining with diaminobenzidine (DAB, Gene Tech) and hematoxylin. Images were obtained with a KFBIO Digital Slide Viewer.

### 2.4. Production of HPV16 E7 Polyclonal Antibody

HPV16 E7 was amplified from HPV16 whole virus plasmids and cloned into the plasmid pGEX4T-2 to obtain plasmid pGEX-4 T-2-HPV16 E7. HPV16 E7 expression was induced in *Escherichia coli* DH5*α* grown in Luria-Bertani (Beyotime, China) medium supplemented with 100 mg/L ampicillin (Beyotime, China) using 0.2 mM isopropyl *β*-D thiogalactopyranoside (Biosharp, China), followed by incubation at 26°C for 3 h. Cells were lysed by ultrasonication (Scientzbio, China) on ice at 200 W for 3 min (5 s on and 10s off). The lysates were centrifuged and supernatants were incubated with Glutaphione Sepharose 4B beads (GE Healthcare, USA) at 4°C for 4 h to capture the GST tag-containing proteins. The GST tag was subsequently removed with Thrombin (GE Healthcare, USA; Supplementary Figure 2). Purified HPV16 E7 was used for subcutaneous immunization of female New Zealand white rabbits (3 months old, approximately 2.5 kg). Blood was collected by cardiocentesis and serum was separated for isolation of IgG antibodies with rProteinG Agarose (Invitrogen, USA). The purified polyclonal antibodies were stored in sterile glycerol (50%) at -20°C. The titer and specificity of the antibodies were verified by Western blot analysis.

### 2.5. Cell Culture and Transfection

Normal human epidermal keratinocytes (NHEKs) were cultured in EpiLife culture medium (Gibco, USA) with 10% fetal bovine serum (Gibco, USA) and Human Keratinocyte Growth Supplement (Gibco, USA). Siha cells were cultured in DMEM (Gibco, USA) containing 10% fetal bovine serum (Gibco, USA). C33A cells were cultured in MEM (Gibco, USA) with 10% fetal bovine serum (Gibco, USA) and 1% Penicillin-Streptomycin Solution (Gibco, USA). All cells were cultured at 37°C in the presence of 5% CO2. Cells at 80% confluence were transfected with GFP-LC3 or mCherry-GFP-LC3 plasmid using Lipofectamine 3000 reagent (Invitrogen, USA). The culture medium was replaced 6 h after transfection and the cells were cultured for another 48 h before analysis.

### 2.6. Construction of HPV16 E7-NHEK Cell Line

The HPV16 E7-expressing NHEK cell lines were constructed by lentivirus infection. The HPV16 E7 gene was cloned into a p23 lentivirus-expression vector and transfected to 293 T cells. The coding sequence of HPV16 E7 is ATGCATGGAGATACACCTACATTGCATGAATATATGTTAGATTTGCAACCAGAGACAACTGATCTCTACTGTTATGAGCAATTAAATGACAGCTCAGAGGAGGAGGATGAAATAGATGGTCCAGCTGGACAAGCAGAACCGGACAGAGCCCATTACAATATTGTAACCTTTTGTTGCAAGTGTGACTCTACGCTTCGGTTGTGCGTACAAAGCACACACGTAGACATTCGTACTTTGGAAGACCTGTTAATGGGCACACTAGGAATTGTGTGCCCCATCTGTTCTCAGAAACCATAA, which was amplified with the forward primer GGCGCGGCCGCATGCATGGAGATACACCTAC and the reverse primer CTAGTCTAGACTCACTTGTCATCGTCGTCCTTGTAGTCTGGTTTCTGAGAACAGATGG, containing a FLAG-tag and XbaI restriction enzyme site. After 48 h, supernatant was collected and centrifuged to get the lentiviral particles. NHEKs were incubated with appropriate lentiviral particles in 12-well plates at a cell density of 1 × 10^5^ with 10 *μ*g/ml polybrene transfection reagent (#TR-1003-G, Merck Millipore) for 8 h, after which the supernatant was replaced with fresh culture medium. Subsequently, the cells were cultured for another 48 h before transfection efficiency was observed by microscopy and flowcytometry. New stable cell lines were established with further flowcytometry sorting. The RNA and protein levels of HPV16 E7 were tested by quantitative real-time PCR and western blot, respectively (Supplementary Figure 3).

### 2.7. Knock-down of DUSP5 and HPV16 E7

NHEKs and Siha were seeded in 6-well plates and incubated for 24 h before transfection with short interfering RNAs (siRNAs). siRNAs targeting three different regions of the DUSP5 or HPV16 E7 gene (siDUSP5 or si-HPV16 E7) and negative control siRNA were synthesized by GenePharma (Shanghai, China). The target sequences for the negative control were UUCUCCGAACHUGUCACGUTT and ACGUGACACGUUCGGAGAATT, while the three pairs of target sequences for DUSP5 were GCUCUUCACUGAUAGGCCATT and UGGCCUAUCAGUGAAGAGCTT,CCAUCUGCAUGGCUUACCUTT and AGGUAAGCCAUGCAGAUGGTT, and GCUCCUGCAGUACGAAUCUTT and AGAUUCGUACUGCAGGAGCTT. Furthermore, three pairs of target sequences for HPV16 E7 were GCAACCAGAGACAACUGAUTT and AUCAGUUGUCUCUGGUUGCTT, GGACAGAGCCCAUUACAAUTT and AUUGUAAUGGGCUCUGUCCTT, and GCUUCGGUUGUGCGUACAATT and UUGUACGCACAACCGAAGCTT. Transfection was performed using Lipofectamine RNAiMAX (Invitrogen, USA) at 70% confluence according to manufacturer's guidelines. 6 h after transfection, the medium was replaced with fresh culture medium. Cells were analyzed at 48 h post transfection for protein expression by Western blotting and quantitative real-time PCR to assess siRNA silencing efficiency.

### 2.8. Western Blotting and Quantitative Real-Time PCR

Experiments were performed as previously described [22]. Antibodies specific for ERK (#4695), JNK (#9252), p38 (#9212), phospho-ERK (Thr202/Tyr204) (#4370), phospho-JNK (Thr183/Tyr185) (#4668), phospho-p38 (Thr180/Tyr182) (#9211), LC3B (#2775S), mTOR (#2983), phospho-mTOR (Ser2448) (#2971), ULK1 (#8054), phospho-ULK1 (Ser555) (#5869), phospho-ULK1 (Ser757) (#14202), phospho-70S6 kinase (Thr389) (#9234), 70S6 kinase(#2708), phospho-4E-BP1 (Thr37/46) (#2855), 4E-BP1 (#9452), AMPK (#2603) and phospho-AMPK (Thr172) (#2531) were purchased from Cell Signaling Technology (Danvers, MA). Antibodies against DUSP5 (#ab200708) were from Abcam (Cambridge, MA) and antibodies against p62 (#PM045) were from MBL Life science. Specific inhibitors U0126 (#HY-12031A), SB203580 (#HY-10256), SP600125 (#HY-12041) and bafilomycin A1 (#HY-100558) were obtained from MCE. Rapamycin (#V900930-1MG) and rose bengal (#330000) were from Sigma. U0216 (10 *μ*M, 24 h), SB203580 (20 *μ*M, 24 h), SP600125 (15 *μ*M, 24 h), bafilomycin A1 (25 nM, 6 h) and rapamycin (100 nM, 12 h) were added to the cell cultures as described in different experiments. The primers for DUSP5 were TGTCGTCCTCACCTCGCTA and GGGCTCTCTCACTCTCAATCTTC.

### 2.9. Immunofluorescence and Confocal Microscopy

Cells were washed with PBS and fixed with 4% paraformaldehyde (Solarbio, China) for 20 min at room temperature. The fixed cells were permeabilized with 0.5% (v/v) Triton X-100 (Solarbio, China) for 10 min, and blocked with 10% goat serum in 1% bovine serum albumin for 1 h at room temperature. HPV16 E7-expressing NHEKs were immunostained with DUSP5 (Absin, #ab200708) while DUSP5-deficient NHEKs were immunostained with LC3B (CST, #2775S) antibodies overnight at 4°C. Subsequently, cells were incubated with Alexa Fluor 594 donkey anti-rabbit IgG (Yeasen, China) for 1 h at 37°C and then stained with 4,6-diamidino-2-phenylindole (DAPI, 1 : 1000 dilution, Abcam) for 10 min at room temperature in the dark. Images were obtained using a Nikon A1 confocal microscope.

### 2.10. GEO Data Mining

Clinical data was retrieved from the Gene Expression Omnibus (GEO) public database. 24 normal cervix and 19 HPV16 positive cervical cancer tissues from the dataset GSE9750 [33], which aimed to identify gene expression profiles in cervical cancer and the role of specific genes in cervical carcinogenesis, were selected. The expression data for DUSP5 in different samples was extracted and analyzed with GEO2R. GEO2R is an open, public and interactive web tool which helps identify gene expression under experimental conditions. See detailed information of each sample in Supplementary Table 1.

### 2.11. Statistical Analysis

For all experiments, at least three biologically independent replicates were performed and the results were analyzed using ImageJ and GraphPad prism7. Splitting the color channel, setting the threshold and size of particles, and analyzing were the main steps for quantification of confocal results. The data are presented as the means ± SEM (standard error of the mean). Statistical analyses include the paired *t*-test or 2-way ANOVA with Dunnett's multiple comparison test. Differences between groups were assessed by Student's *t*-test and p <0.05 was considered statistically significant. ∗, p <0.05; ∗∗, p <0.01; ∗∗∗, p <0.005; ∗∗∗∗, p <0.001.

## 3. Results

### 3.1. Inverse Correlation between HPV16 E7 and DUSP5 Expression

**Figure 1:**
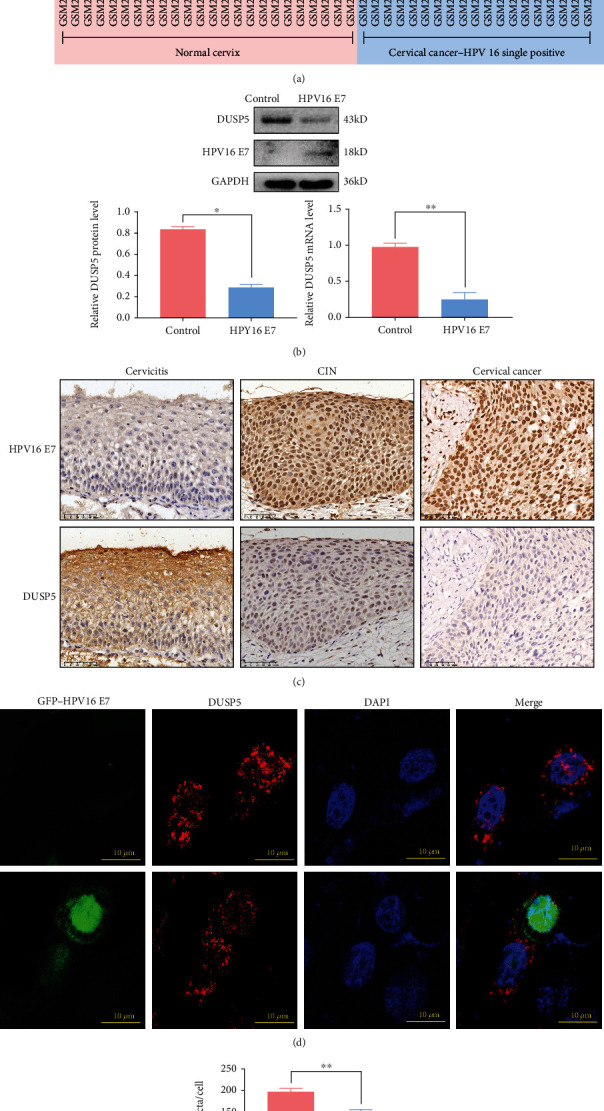
Inverse correlation between HPV16 E7 and DUSP5. (a) Expression levels of DUSP5 (GEO dataset GSE9750) in normal cervical epithelium and HPV16 single positive cervical cancer tissues. (b) Western analysis and quantitative real-time PCR analysis of DUSP5 in control and HPV16 E7-expressing NHEKs. (c) Immunohistochemical staining of HPV16 E7 and DUSP5 in cervicitis, cervical intraepithelial neoplasia (CIN) and cervical cancer tissues. (d) Confocal microscopy analysis of HPV16 E7 (Green) and DUSP5 (Red) in HPV16 E7-expressing NHEKs. (e) Quantification of DUSP5 puncta from confocal microscopy images. The graph represents the mean and standard deviation from three biological independent repeats, and at least 10 cells were analyzed per repeat. ImageJ was used for quantification. Significant differences were identified by Student's *t*-test. ∗, P <0.05. ∗∗, p <0.01.

In our previous study we transfected a HPV16 E7-expression vector into NHEKs and analyzed its effect on global transcriptional profiles. Pathway analysis showed differential gene expression of clusters associated with MAPK signaling, with specific downregulation of DUSP5 in HPV16 E7-positive NHEKs. To investigate the correlation between HPV16 E7 and DUSP5 gene expression in human clinical samples, the GSE9750 dataset available in GEO, which contains expression data of HPV16-positive human cervical cancer and control tissues, was analyzed. Indeed, gene expression levels of DUSP5 were significantly lower in HPV16 single positive cervical cancer tissues compared with normal cervical control tissues (Figure 1(a) and Supplementary Table 1), indicating a possible inverse correlation between HPV16 E7 and DUSP5. To further validate an inverse correlation between HPV16 E7 and DUSP5 at both mRNA and the protein levels, we used a lentivirus expression system for stable expression of E7 in NHEKs. Subsequent analysis of DUSP5 in E7-expressing NHEKs showed a significant reduction in DUSP5 protein and gene expression levels compared with control NHEKs (Figure 1(b)). Importantly, the inverse correlation between HPV16 E7 and DUSP5 was also observed in human cervical tissues. While HPV16 E7-negative cervicitis tissues showed high DUSP5 levels, HPV16 E7-positive cervical intraepithelial neoplasia (CIN) and cervical cancer tissues showed low DUSP5 expression levels (Figure 1(c)), indicating that DUSP5 plays an important role in disease progression. Finally, immunostained DUSP5 was detected by confocal microscopy in HPV16 E7-expressing NHEKs (Figure 1(d)). Compared with NHEK control cells, DUSP5 expression was significantly lower in both the nucleus and cytoplasm of HPV16 E7-positive NHEKs (Figure 1(e)). Overall, these results indicate that HPV16 E7 reduces DUSP5 expression levels.

### 3.2. HPV16 E7-Mediated DUSP5 Deficiency Induces Autophagy Flux and Autophagosome Formation

**Figure 2:**
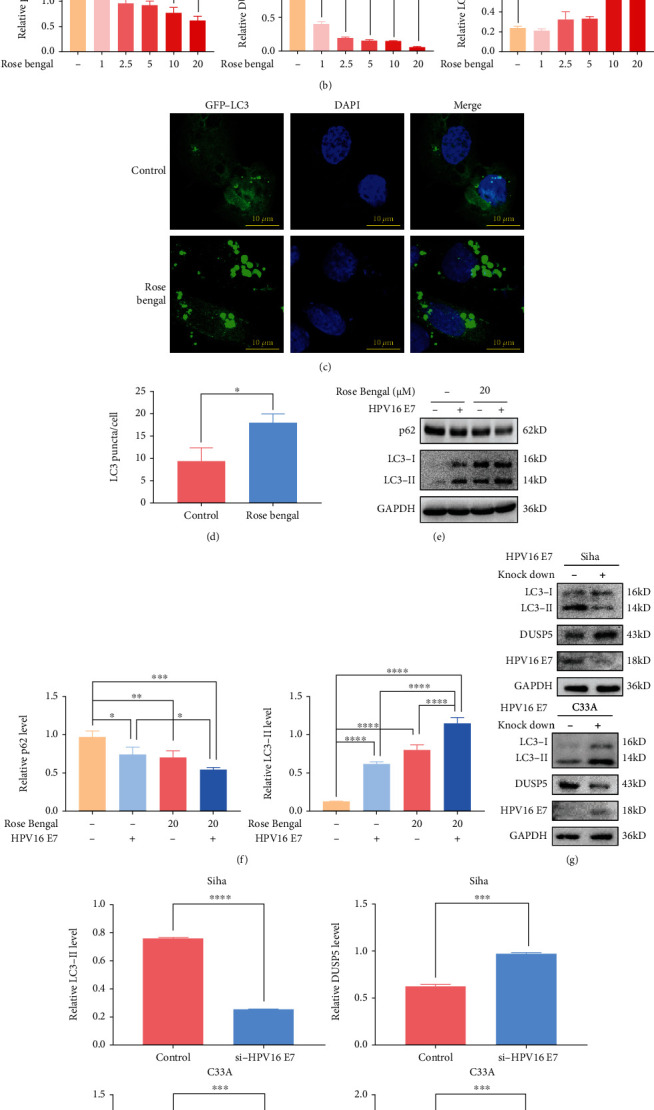
HPV16 E7-mediated DUSP5-deficiency induces autophagy. (a) Western analysis of p62, DUSP5 and LC3 levels in NHEKs after incubation with increasing concentrations of rose bengal (0 to 20 *μ*M) for 48 h. (b) Quantification of p62, DUSP5 and LC3-II levels from panel a, using GAPDH for normalization. (c) Representative confocal microscopy images of NHEKs expressing GFP-LC3 with or without treatment with 20 *μ*M rose bengal. (d) Quantification of LC3 puncta per cell from confocal microscopy images in panel c. The graph represents the mean and standard deviation from three biological independent repeats, and at least 10 cells were analyzed per repeat. (e) Western analysis of p62 and LC3 levels in HPV16 E7-expressing or control NHEKs after rose bengal treatment (0 or 20 *μ*M, 8 h). (f) Quantification of p62 and LC3-II levels from panel e, using GAPDH for normalization. (g) Western analysis of LC3, DUSP5 and HPV16 E7 levels in HPV16-expressing Siha cells after knock-down of E7 and in C33A cells after expression of HPV16 E7. (h) Quantification of LC3-II and DUSP5 levels from panel g, using GAPDH for normalization. ImageJ was used for quantification. Significant differences were identified by Student's *t*-test. ∗, p <0.05; ∗∗, p <0.01; ∗∗∗, p <0.005; ∗∗∗∗, p <0.001.

Given the role of DUSPs in regulation of MAPK signaling and consequently autophagy activity, we investigated whether HPV16 E7-mediated DUSP5 deficiency activates autophagy. We incubated NHEKs with increasing concentrations of rose bengal, which is an FDA-approved compound with strong potency against DUSP5 [34], and showed by Western analysis that DUSP5 protein levels were indeed decreased in a rose bengal concentration-dependent manner (Figures 2(a) and 2(b)). We furthermore investigated its impact on the autophagy activity marker proteins LC3 and p62. The expression levels of LC3-II, which is an essential component of the phagophore, were significantly higher after rose bengal treatment, while the levels of p62, which is a receptor for autophagy cargos, were significantly lower at higher doses. Similarly, rose bengal treatment of NHEKs transfected with a plasmid expressing GFP-LC3 showed a significant increase in LC3 puncta compared with control cells (Figures 2(c) and 2(d). Interestingly, while expression of HPV16 E7 in NHEKs resulted similarly in an elevation of LC3-II levels and reduction of p62 levels as observed after rose bengal treatment, combining rose bengal treatment with HPV16 E7 expression appears to provide additive autophagy responses, given that LC3-II levels were further increased in the combined treatment and p62 levels further reduced (Figures 2(e) and 2(f)). To validate the impact of HPV16 E7 on DUSP5 expression and autophagy in other cell models, E7 was knock-down in HPV16-positive Siha cells or expressed in the HPV-negative C33A cells. Subsequent Western analysis showed an increase in DUSP5 levels and decrease of LC3-II levels after E7 knock-down in Siha cells, while DUSP5 was decreased and LC3-II increased after expression of HPV16 E7 in C33A cells (Figures 2(g) and 2(g)).

**Figure 3:**
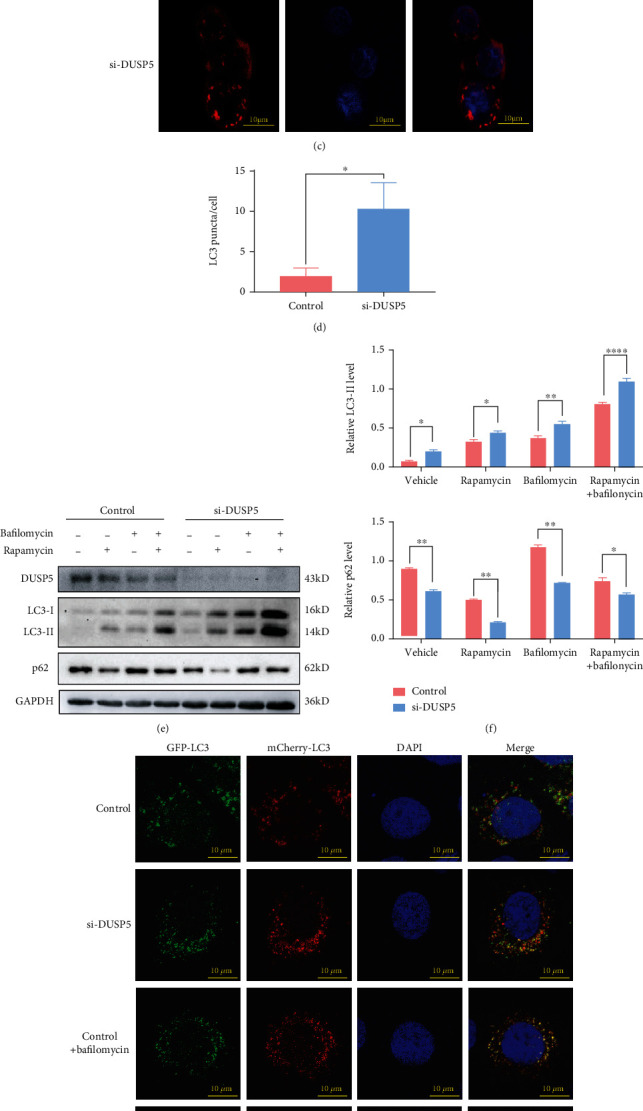
DUSP5 deficiency induces autophagy flux and autophagosome formation. Western analyses of DUSP5, LC3 and p62 levels in DUSP5-deficient and control NHEKs. (b) Quantification of DUSP5, LC3-II and p62 levels from panel a, using GAPDH for normalization. (c) Representative confocal microscopy images of endogenous immunostained LC3 puncta in DUSP5 deficient and control NHEKs. (d) Quantification of LC3 puncta per cell from panel c. (e) Western analysis of DUSP5, LC3 and p62 levels in DUSP5 deficient and control NHEKs after treatment with 100 nM rapamycin for 12 h or 25 nM bafilomycin A1 for 6 h. (f) Quantification of LC3-II and p62 levels from panel e, using GAPDH for normalization. (g) Representative confocal microscopy images of vehicle or BafA1 pretreated DUSP5 deficient and control NHEKs transfected with a vector expressing mCherry-GFP-LC3. (h) Quantification of the ratio of red LC3 puncta (autolysosomes) to all LC3 puncta (autophagosomes plus autolysosomes) from images represented in panel g. The graph represents the mean and standard deviation from three biological independent repeats, and at least 10 cells were analyzed per repeat. ImageJ was used for quantification. Significant differences were identified by Student's *t*-test. ∗, p <0.05; ∗∗, p <0.01; ∗∗∗, p <0.005; ∗∗∗∗, p <0.001.

To elaborate on these results, we knocked-down DUSP5 in NHEKs using siRNA transfection and again investigated LC3-II and p62 levels. The expression levels of LC3-II were significantly higher after knock-down of DUSP5, while the levels of p62 were significantly lower (Figures 3(a) and 3(b)). Furthermore, confocal microscopy analysis of immunostained LC3 showed higher numbers of LC3 puncta per cell, a hallmark of autophagosome biogenesis, in DUSP5 knock-down cells compared with control cells (Figures 3(c) and 3(d)). Therefore, these results indicate that DUSP5-deficient NHEKs display increased numbers of autophagosomes that are able to sequester p62-loaded cargos. To further investigate whether the increase in autophagosomes in DUSP5-deficient NHEKs was the result of increased autophagy activity or inhibited recycling, autophagy flux was analyzed using the canonical autophagy activator rapamycin and the V-ATPase inhibitor bafilomycin A1 (BafA1), which inhibits autophagosome-lysosome fusion. Addition of rapamycin, BafA1 or a combination of these increased the levels of LC3-II, with higher levels observed in DUSP5 knock-down NHEKs compared with control cells (Figures 3(e) and 3(f)). Furthermore, addition of rapamycin resulted in a decrease in p62 levels in both control and knock-down cells, which was prevented by addition of BafA1. Finally, autophagosome maturation into autolysosomes was investigated after transfection with a plasmid expressing an mCherry-GFP-LC3 fusion protein. Since GFP is acid sensitive, its fluorescent activity is lost in the acidic environment of the autolysosome. Therefore, autophagosomes that mature into autolysosomes are visible as red puncta. Indeed, more red puncta were observed in DUSP5 deficient NHEKs than in control cells (Figures 3(g) and 3(h)), indicating that autophagosome maturation in DUSP5-deficient NHEKs was unaffected. As a control, cells were treated with BafA1, which inhibited autolysosome formation in both DUSP5-deficient NHEKs and control cells. Collectively, these data suggest that DUSP5 deficiency induces autophagy fluxes and does not interfere in autophagosome biogenesis and maturation processes.

### 3.3. DUSP5 Inhibits Autophagy via the MAPK/ERK Pathway

**Figure 4:**
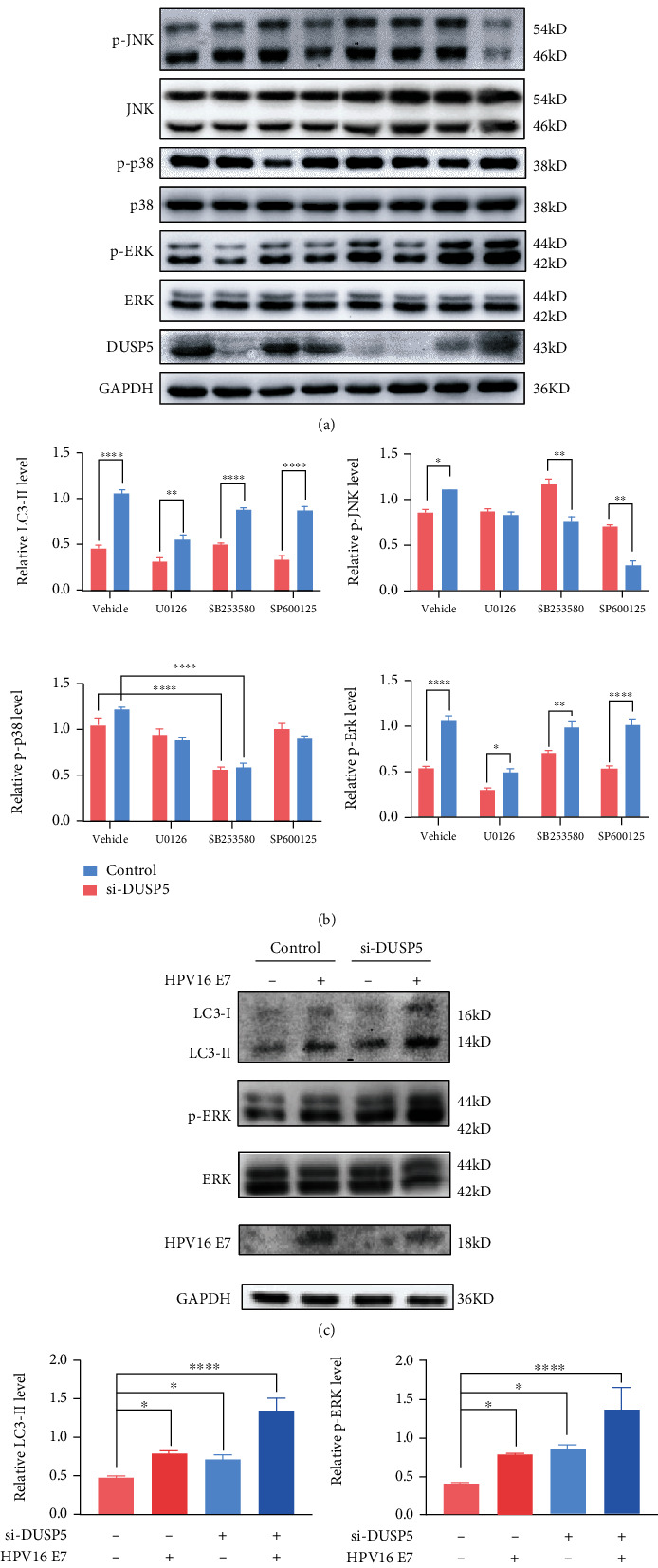
DUSP5 deficiency activates autophagy via the MAPK/ERK pathway. (a) Western analysis of phosphorylated and total JNK, p38, and ERK levels and DUSP5 and LC3 levels in DUSP5-deficient and control NHEKs. Cells were pretreated with U0126 (10 *μ*M), SB203580 (20 *μ*M), SP600125 (15 *μ*M) or a vehicle control for 24 h. (b) Quantification of LC3-II and phosphorylated JNK, p38, and ERK levels from panel a, using GAPDH (LC3-II) or total JNK, p38, and ERK levels for normalization. (c) Western analysis of LC3, HPV16 E7, and phosphorylated and total ERK levels in HPV16 E7-expressing DUSP5-deficient NHEKs or control cells. (d) Quantification of LC3-II and phosphorylated ERK levels from panel c, using GAPDH (LC3-II) or total ERK levels for normalization. Significant differences were identified by Student's *t*-test. ∗, p <0.05; ∗∗, p <0.01; ∗∗∗, p <0.005; ∗∗∗∗, p <0.001.

To explore the mechanism by which DUSP5 modulates autophagy activity, DUSP5-deficient and control NHEKs were investigated for the phosphorylation status of ERK, JNK and p38 in the presence and absence of inhibitors U0126 (p-ERK), SB203580 (p-p38) or SP600125 (p-JNK) after treatment for 24 h (Figures 4(a) and 4(b)). The levels of p-ERK were most significantly elevated in DUSP5-deficient NHEKs compared with control cells, while p-JNK showed a moderate but significant increase. Furthermore, addition of U0126 significantly reduced p-ERK levels in both DUSP5-deficient and control NHEKs, but a significant difference between these in p-ERK remained, which indicates that U0126 and DUSP5 do not interfere in each other's p-ERK-modulating activity. Importantly, only addition of U0126 and not addition of SB203580 or SP600125 resulted in a significant reduction in LC3-II levels, indicating that in NHEKs, DUSP5 deficiency activates autophagy fluxes by inducing the MAPK/ERK pathway. Interestingly, the p-JNK inhibitor seemed to elevate DUSP5 levels in the DUSP5 knock-down NHEKs, while the levels of p-JNK were most severely reduced in SP600125-treated DUSP5 knock-down cells. These results may be supported by the previously demonstrated feedback cross-regulation between JNK and ERK signaling cascades and DUSP5 [35]. Finally, the role of HPV16 E7 in DUSP5-deficiency-mediated increased p-ERK levels was validated. NHEKs expressing HPV16 E7 showed increased p-ERK levels compared with control cells (Figures 4(c) and 4(d)) and combining HPV16 E7 expression with DUSP5 siRNA was further cumulative for p-ERK levels.

### 3.4. DUSP5 Regulates Autophagy through mTOR Signaling

**Figure 5:**
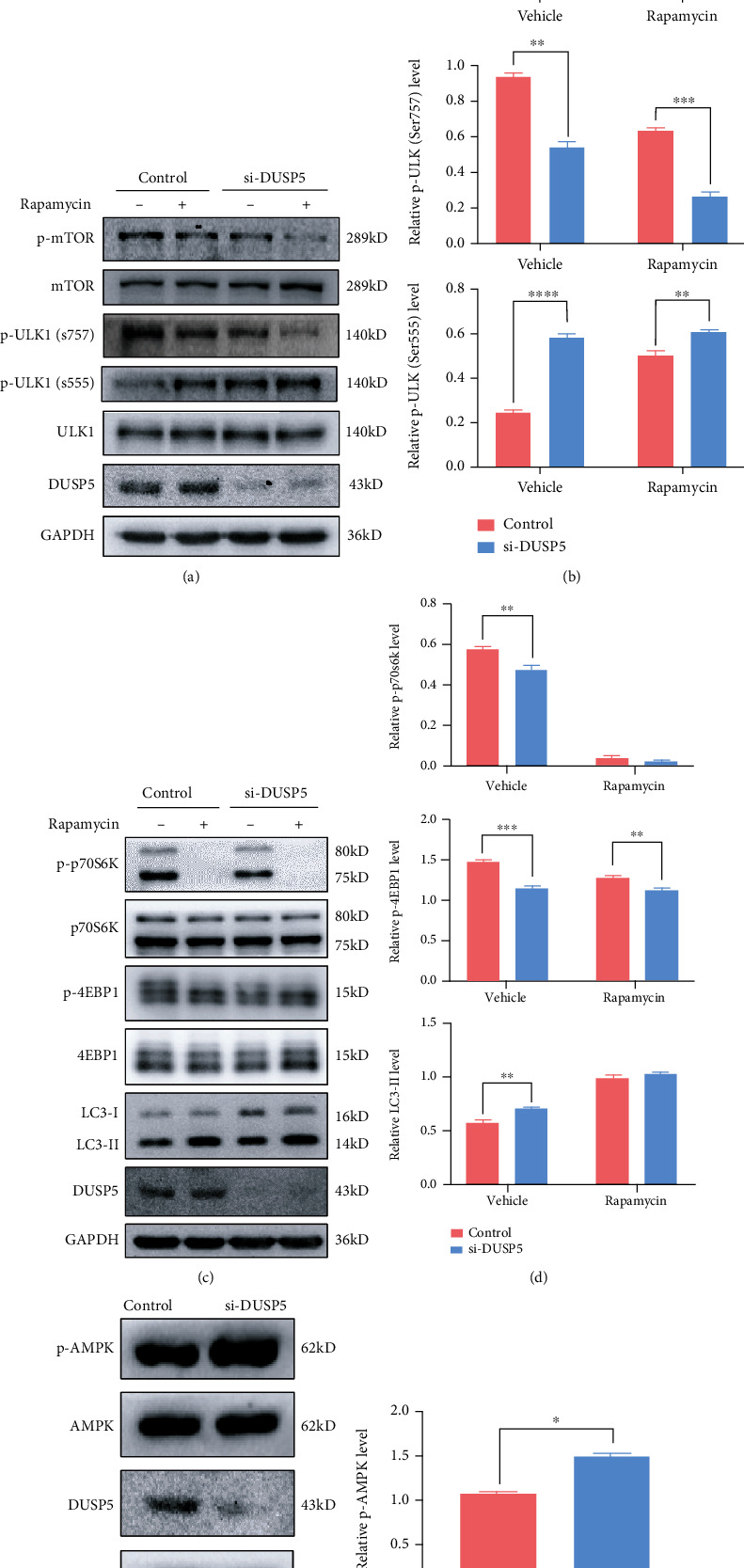
Effects of DUSP5 deficiency on mTOR signaling. (a) Western analysis of phosphorylated and total mTOR and ULK1 levels and DUSP5 levels inDUSP5-deficient and control NHEKs after pre-treatment with rapamycin or a vehicle control. (b) Quantification of phosphorylated mTOR and ULK1 levels from panel a, using total mTOR and ULK1 levels for normalization. (c) Western analysis of phosphorylated and total P70S6K and 4EBP1 levels and LC3 and DUSP5 levels in DUSP5-deficient and control NHEKs after pre-treatment with rapamycin or a vehicle control. (d) Quantification of phosphorylated P70S6K and 4EBP1 levels and LC3-II levels from panel c, using GAPDH (LC3-II) or total P70S6K and 4EBP1 levels for normalization. (e) Western analysis of phosphorylated and total AMPK levels and DUSP5 levels in DUSP5-deficient and control NHEKs. (f) Quantification of phosphorylated AMPK levels in panel e, suing total AMPK levels for normalization. Significant differences were identified by Student's *t*-test. ∗, p <0.05; ∗∗, p <0.01; ∗∗∗, p <0.005; ∗∗∗∗, p <0.001.

Mammalian target of rapamycin (mTOR) is activated as part of mTOR complex 1 (mTORC1) and suppresses canonical autophagy by phosphorylating ULK1 at serine 757, while phosphorylation of ULK1 at serine 555 by AMP activated protein kinase (AMPK) results in activation of autophagy [36]. In addition, activated mTORC1 phosphorylates its downstream substrates 4EBP1 and p70S6K, which are not related to autophagy. HPV16 E7 activates autophagy through mTOR signaling in NHEKs (Supplementary Figure 4). To further explore the mechanism by which DUSP5 deficiency promotes autophagy, the phosphorylation state of mTOR, ULK1, p70S6K, 4EBP1 and AMPK was analyzed in DUSP5-deficient and control NHEKs. Phosphorylation of mTOR, ULK1 (S757), p70S6K and 4EBP1 was significantly reduced in DUSP5-deficient NHEKs compared with control cells (Figures 5(a) and 5(d)). Addition of rapamycin further reduced phosphorylation levels. In contrast, phosphorylation of ULK1 (S555) and AMPK was promoted in DUSP5-deficient NHEKs (Figures 5(a), 5(b), 5(e) and 5(f)). Therefore, our results show that DUSP5 deficiency activates canonical autophagy through suppression of mTOR and activation of AMPK, resulting in activation of ULK1.

## 4. Discussion

Persistent HR-HPV infection is the leading cause of cervical cancer worldwide. The oncogenic effects of HPV are closely related to expression of oncoprotein E7, which is involved in the modulation of cell proliferation, apoptosis, and immortality. In response to metabolic stress, autophagy is crucial for survival and maintenance of cellular nutrient homeostasis. Previous research has shown that autophagy is inhibited at the early stages during cellular entry [37], but reactivated at the later stages of HPV-related anal cancer, as shown by detection of autophagy markers in low-grade and high-grade squamous intra-epithelial lesions [38, 39]. In our previous studies, we found that both LR-HPV type 11 pseudovirions and LR-HPV early protein E6 activate autophagy through ERK/mTOR and Akt/mTOR signaling [40, 41]. Although host cell-induced autophagy may target and eliminate invading viruses such as HPV, active autophagy fluxes are required to meet the tumorigenic potential and growing demand of cancer cell metabolism in advanced HPV infection stages. Therefore, tight regulation of autophagic activity is likely important during the HPV infection cycle and could be a specific target for treatment of cervical cancer and development of new prophylactic or therapeutic antiviral strategies.

In our previous study, we explored the cellular targets of the HPV16 E7 oncoprotein to better understand vital cellular signal transduction pathways in the modulation of cellular homeostasis and identified its repressive activity to DUSP5. Here, we now show that HR-HPV16 E7 promotes autophagy fluxes through DUSP5 repression, which promotes ERK/mTOR and AMPK signaling. Luo et al. also found an inverse correlation between HPV16 E7 and STING protein levels and loss of HPV16 E7 led to reduced autophagy. That study furthermore showed that HPV16 E7 promotes autophagy-dependent degradation of STING, which suppresses STING-dependent IFN-I responses [42]. Mattoscio et al. found enriched LC3 in autophagic structures and accumulation of p62 in HPV16 E6/E7-expressing primary human keratinocytes by transmission electron microscopy and Western blotting, suggesting that autophagosome-lysosome fusion was blocked. Furthermore, by comparing empty vector, E6, E7 and HPV16 E6/E7-transduced keratinocytes, they concluded that the blockage of late steps in basal autophagy was E6-dependent, because accumulation of p62 was already observed after transduction with HPV16 E6 alone [43]. Activated autophagy has been confirmed to correlate with poor prognosis in some cancer types, while targeting cancer cells with autophagosome inhibitor 3-methyl adenine induced apoptosis, pointing to a possible anticancer therapy [44].

Some studies have shown that activated MAPK/ERK signaling is involved in tumor growth and proliferation in cervical cancer [45, 46]. Therefore, targeting DUSPs, which are key regulators of MAPKs, is expected to be a suitable strategy for anticancer drug development. Our current study showed that expression of DUSP5 is severely repressed in cervical cancer tissues. Consistent with our results, DUSP5 expression in pre-eclampsia inhibits trophoblast proliferation, migration and invasion [47]. In addition, it has been shown that DUSP5-mediated ERK suppression can serve as a protective mechanism by blocking pulmonary vascular smooth muscle cell proliferation, by preventing pulmonary hypertension and right ventricular cardiac hypertrophy [48] and by inhibiting inflammation in adipocytes [35]. It has furthermore been shown that DUSP5 is critical for regulation of immunity and tolerance, since DUSP5-expressing mature T cells exhibit decreased IL-2-dependent proliferation and defective IL-2-mediated induction of genes [49]. Collectively, these studies suggest that DUSP5 has important functions in mammalian biology and may serve as a viable drug target.

Increased ERK phosphorylation was found in neural progenitor cells [50] and the rat fibroblast cell line 3Y1 [51], and human keratinocytes expressing HPV oncogene E7 or inhibitors of ERK signaling can reduce oncogene expression and inhibit a neoplastic phenotype through EGFR/MEK/ERK signaling [52] or K-Ras/ERK signaling [53, 54]. In a skin carcinogenesis mouse model, papilloma formation was found in mice lacking DUSP5 and its regulation of nuclear ERK activity also served DUSP5 as tumor suppressor in epidermis Ras modulation [55]. We consistently found DUSP5 deficiency increased ERK phosphorylation, similar as observed after HPV16 E7 expression. mTOR signaling was also found to be regulated by DUSP5, and based on their studies by Bermudez et al. on DUSP6 [56] and Benavides-Serrato et al. on DUSP10 and the mTORC2 pathway in glioblastoma [57], there might be crosstalk between the ERK and mTOR pathways. These studies and our result highlight the future potential of modulating ERK signaling for HPV neoplasia treatment.

## 5. Conclusions

**Figure 6:**
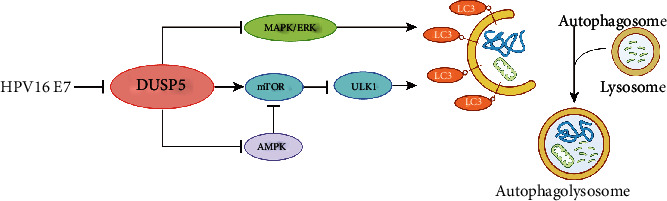
Schematic diagram showing that HPV16 E7 induces autophagy by inhibiting DUSP5 through the MAPK/ERK pathway and mTOR signaling.

In conclusion, understanding the role of autophagy during different stages of HPV-associated diseases provides an opportunity to generate novel autophagy-targeting treatment strategies. Our report sheds light on a novel molecular mechanism explaining how HPV16 E7 modulates host-cell autophagy, an important pathway for HPV-mediated malignant transformation and disease. We demonstrate that DUSP5 expression is inversely correlated with HPV16 E7 expression and that HPV16 E7-mediated repression of DUSP5 induces autophagy via phosphorylation of MAPK/ERK, mTOR and AMPK, resulting in activation of ULK1 (Figure 6).

## Data Availability

The datasets used and analyzed during the current study are available from the corresponding author upon reasonable request.

## Abbreviations

HPV: Human papillomavirus
NHEKs: Normal human epidermal keratinocytes.

## Supplementary Materials

Supplementary 1 Supplementary Figure 1: DNA Gel Electrophoresis of nested PCR products. M: DNA marker, Lane1: cervicitis tissue, Lane2: cervical intraepithelial neoplasia (CIN) tissue, Lane3: cervical cancer tissue.

Supplementary 2 Supplementary Figure 2: Verification of HPV16 E7 antibodies. Coomassie blue staining of different products during antibody preparation. M: protein marker, Lane1: supernatant of DH5*α* lysates after ultrasonication, Lane2: precipitation of DH5*α* lysates after ultrasonication, Lane3: supernatant of Lane1 lysates after incubation with Glutaphione-Sepharose 4B beads (GST fusion protein), Lane4: supernatant of Lane3 lysates after incubation with thrombin and cleavage of GST tag (HPV16 E7 protein), Lane5: precipitation of Lane4.

Supplementary 3 Supplementary Figure 3: Verification of HPV16 E7-expressing NHEKs. (a) Fluorescence microscopy observation of NHEKs carrying mock lentivirus and HPV16 E7. (b) Fluorescence activated cell sorting by flow cytometry of negative control, mock and HPV16 E7 lentivirus infected NHEKs. (c) Western analysis of HPV16 E7 in mock and HPV16 E7 lentivirus-infected NHEKs.

Supplementary 4 Supplementary Figure 4: HPV16 E7 activates autophagy through mTOR signaling. (a) Western analysis of phosphorylated and total mTOR, ULK1, P70S6K, and 4EBP1 and LC3 in HPV16 E7-expressing and control NHEKs. (b) Quantification of LC3-II, DUSP5 and phosphorylated mTOR, ULK1, P70S6K, and 4EBP1 levels from panel a, using GAPDH (LC3-II and DUSP5) or total mTOR, ULK1, P70S6K, and 4EBP1 levels for normalization. Significant differences were identified by Student's t-test. ∗, p <0.05; ∗∗.

Supplementary 5 Supplementary Table 1: Accession, characteristics and value of DUSP5 of normal cervix or cervical cancer samples.
