# Supplementary figures and images for: Human Papillomavirus Type 16 Early Protein E7 Activates Autophagy through Inhibition of Dual-Specificity Phosphatase 5
Source: Oxid Med Cell Longev. 2022 Mar 10;2022:1863098. doi: 10.1155/2022/1863098 (PMC8966754; doi:10.1155/2022/1863098)

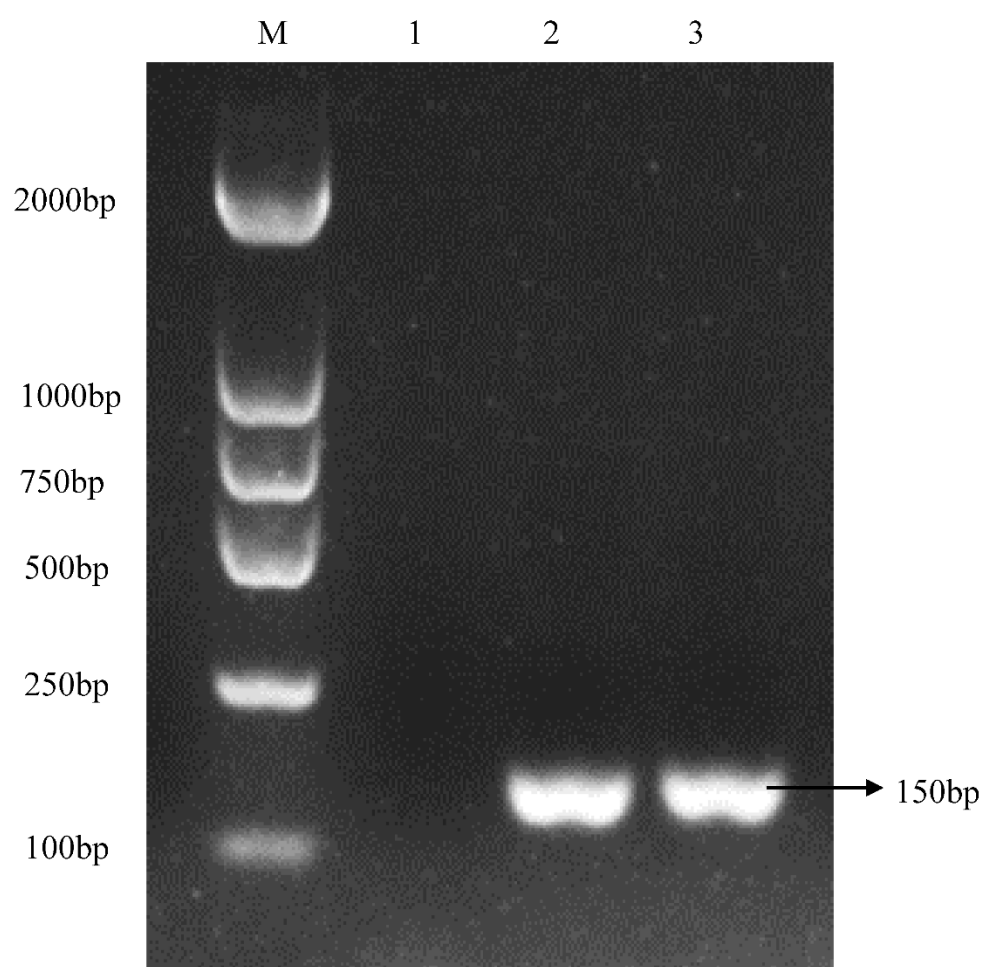

Supplement: Supplementary 1 — Supplementary Figure 1: DNA Gel Electrophoresis of nested PCR products. M: DNA marker, Lane1: cervicitis tissue, Lane2: cervical intraepithelial neoplasia (CIN) tissue, Lane3: cervical cancer tissue. [file 1863098.f1.pdf]

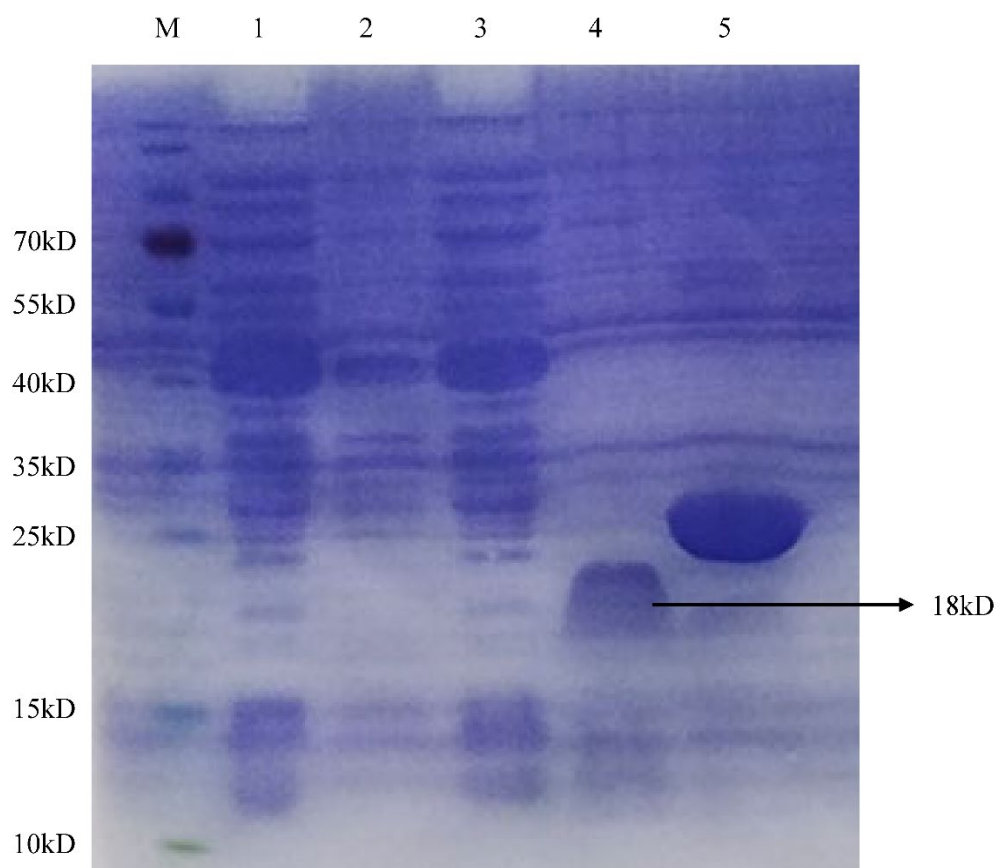

Supplement: Supplementary 2 — Supplementary Figure 2: Verification of HPV16 E7 antibodies. Coomassie blue staining of different products during antibody preparation. M: protein marker, Lane1: supernatant of DH5α lysates after ultrasonication, Lane2: precipitation of DH5α lysates after ultrasonication, Lane3: supernatant of Lane1 lysates after incubation with Glutaphione-Sepharose 4B beads (GST fusion protein), Lane4: supernatant of Lane3 lysates after incubation with thrombin and cleavage of GST tag (HPV16 E7 protein), Lane5: precipitation of Lane4. [file 1863098.f2.pdf]

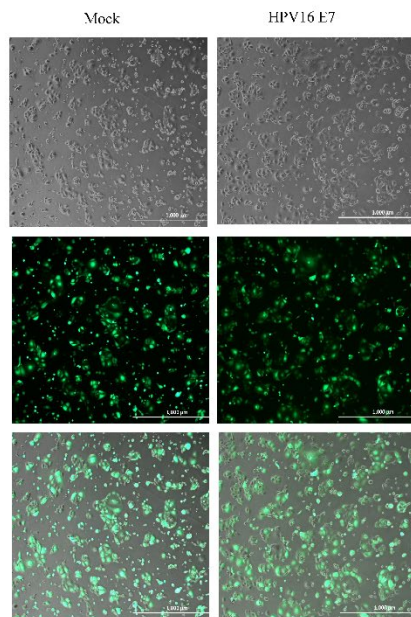

(a)

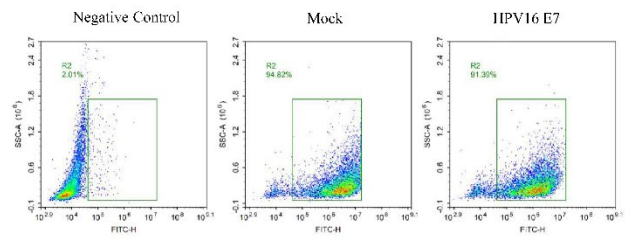

(b)

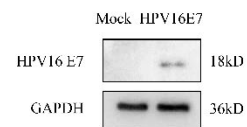

(c)

Supplement: Supplementary 3 — Supplementary Figure 3: Verification of HPV16 E7-expressing NHEKs. (a) Fluorescence microscopy observation of NHEKs carrying mock lentivirus and HPV16 E7. (b) Fluorescence activated cell sorting by flow cytometry of negative control, mock and HPV16 E7 lentivirus infected NHEKs. (c) Western analysis of HPV16 E7 in mock and HPV16 E7 lentivirus-infected NHEKs. [file 1863098.f3.pdf]

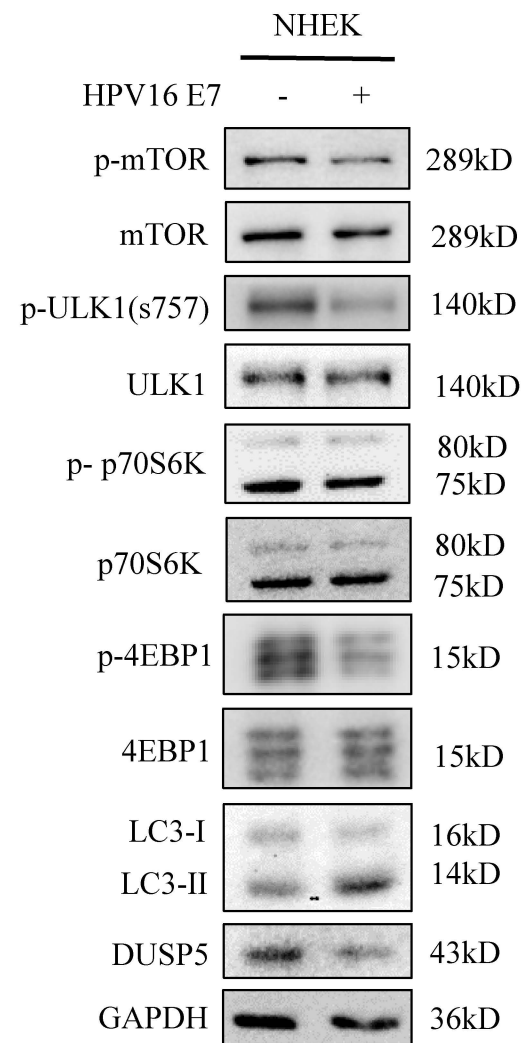

(a)

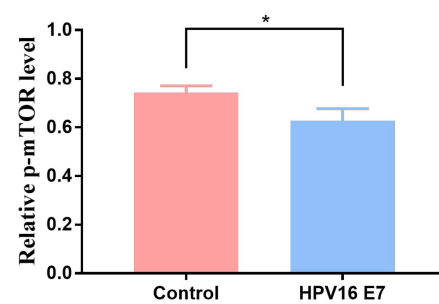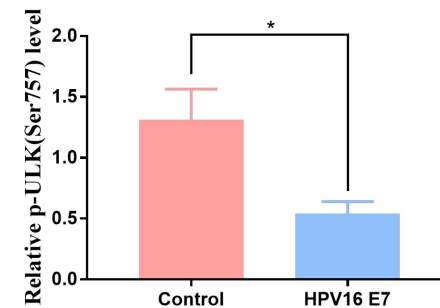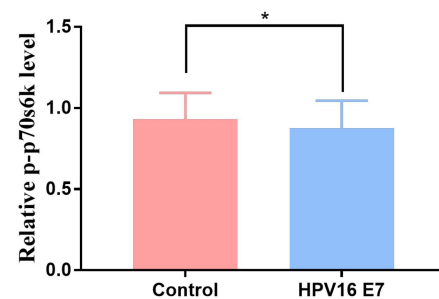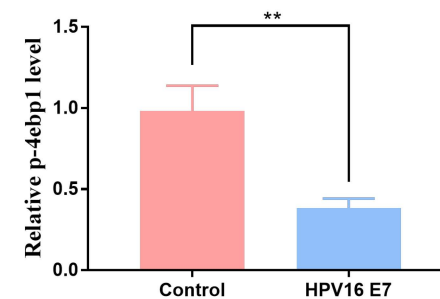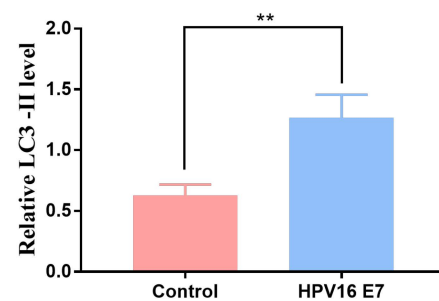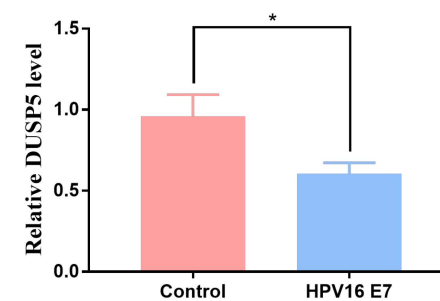

(b)

Supplement: Supplementary 4 — Supplementary Figure 4: HPV16 E7 activates autophagy through mTOR signaling. (a) Western analysis of phosphorylated and total mTOR, ULK1, P70S6K, and 4EBP1 and LC3 in HPV16 E7-expressing and control NHEKs. (b) Quantification of LC3-II, DUSP5 and phosphorylated mTOR, ULK1, P70S6K, and 4EBP1 levels from panel a, using GAPDH (LC3-II and DUSP5) or total mTOR, ULK1, P70S6K, and 4EBP1 levels for normalization. Significant differences were identified by Student's t-test. ∗, p <0.05; ∗∗. [file 1863098.f4.pdf]
