# Supplementary material for: Human Papillomavirus Type 16 Early Protein E7 Activates Autophagy through Inhibition of Dual-Specificity Phosphatase 5
Source: Oxid Med Cell Longev. 2022 Mar 10;2022:1863098. doi: 10.1155/2022/1863098 (PMC8966754; doi:10.1155/2022/1863098)
Supplement: Supplementary 5 — Supplementary Table 1: Accession, characteristics and value of DUSP5 of normal cervix or cervical cancer samples. [file 1863098.f5.pdf]

Accession	Characteristics	Title	Value of DUSP5
GSM246422		Normal cervix, commercial_Ambion	862.4
GSM246423	Toral RNA, commercial	Normal cervix, commercial_Stratagene	492.3
GSM246484		Normal cervix, commercial_BioChain	636.4
GSM246485	Normal cervix, age 27 years, micordissected squamous epitheilium	Normal cervix epithelium_CaCX3	55.1
GSM246486	Microdissected normal cervical epithelium, Age 38	Normal cervix epithelium_CaCx4	556.7
GSM246487	Normal cervical epithelium, microdissected, Age 30	Normal cervix epithelium_CaCx5	177.6
GSM246488	Normal cervix epithelium, microdissected, Age 43	Normal cervix epithelium_03-3505	155.1
GSM246489	Normal cervix, microdissected, Age 42	Normal cervix epithelium_03-4216	1753.2
GSM246490	Normal cervix epiethelium, microdissected, Age 48	Normal cervix epithelium_03-4508	532.8
GSM246491	Normal cervix epithelium, microdissected, Age 42	Normal cervix epithelium_03-4986	1713.3
GSM247162	Normal cervix, microdissected epithelium, Age 45	Normal cervix epithelium_03-5419	345.8
GSM247163	Normal Cervix, microdissected epithelium, Age 54	Normal cervix epithelium_03-5438	1569.1
GSM247164	Normal cervix epithelium, microdissected, Age 44	Normal cervix_03-5611	2272.5
GSM247165	Normal cervix epithelium, Age 49	Normal cervix_03-5657	1046.3
GSM247166	Normal cervix epithelium, Age 64	Normal cervix_05-15	1312.4
GSM247168	Normal cervix epithelium, Age 50	Normal cervix_05-31	796.6

GSM247169	Normal cervix, Age 50	Normal cervix_05-446	1232.7
GSM247171	Normal cervix, Age 56	Normal cervix_05-1308	864.7
GSM247173	Normal cervix epithelium, Age 41	Normal cervix_05-1352	641.7
GSM247174	Normal cervix epithelium, Age 41	Normal cervix_05-1981	3288.1
GSM247175	Normal cervix epithelium, Age 49	Normal cervix_05-4602	1621.3
GSM247188	Normal cervix epithelium, Age 50	Normal cervix_05-4615	3770
GSM247189	Normal cervix epithelium, Age 53	Normal cervix_05-4959	414.4
GSM247190	Normal cervix epithelium, Age 52	Normal cervix_05-5007	482.5
GSM247651	Cervical cancer, squamous cell carcinoma, microdissected, age 55, stage IIIB, HPV 16	Cervical cancer_CC128	268.4
GSM247652	Cervical cancer, squamous cell carcinoma, microdissected, age 41, stage IIIB, HPV 16	Cervical cancer_CC140	119.2
GSM247654	Cervical cancer, squamous cell carcinoma, microdissected, age 45, stage IIIB, HPV 16	Cervical cancer_CC205	133
GSM247655	Cervical cancer, squamous cell carcinoma, microdissected, age 54, stage IIIB, HPV 16	Cervical cancer_CC207	394.2
GSM247656	Cervical cancer, squamous cell carcinoma, microdissected, age 43, stage IIB, HPV 16	Cervical cancer_CC214	99.3
GSM247657	Cervical cancer, squamous cell carcinoma, microdissected, age 40, stage IIIB, HPV 16	Cervical cancer_CC218	403.4

GSM247658	Cervical cancer, squamous cell carcinoma, microdissected, age 32, stage IIIB, HPV 16	Cervical cancer_CC222	228.4
GSM247659	Cervical cancer, squamous cell carcinoma, microdissected, age 40, stage IIIB, HPV 16	Cervical cancer_892T	852.4
GSM247661	Cervical cancer, squamous cell carcinoma, microdissected, age 33, stage IIB, HPV 16	Cervical cancer_1798T	1164
GSM247662	Cervical cancer, squamous cell carcinoma, microdissected, age 36, stage IIB, HPV 16	Cervical cancer_1875T	894.1
GSM247857	Cervical cancer, squamous cell carcinoma, microdissected, age 58, stage IIA, HPV 16	Cervical cancer_841T	426.9
GSM247859	Cervical cancer, squamous cell carcinoma, microdissected, age 47, stage IIB, HPV 16	Cervical cancer_1434T	399.9
GSM247860	Cervical cancer, squamous cell carcinoma, microdissected, age 69, stage IIB, HPV 16	Cervical cancer_1509T	139.3
GSM247865	Cervical cancer, squamous cell carcinoma, microdissected, age 33, stage IV, HPV 16	Cervical cancer_1907T	654.4
GSM247876	Cervical cancer, adenocarcinoma, microdissected, age 46, stage IB2, HPV 16	Cervical cancer_20-04T	119.4

GSM247881	Cervical cancer, squamous cell carcinoma, microdissected, stage IIIA, HPV 16	Cervical cancer_103-04T	465.2
GSM247882	Cervical cancer, squamous cell carcinoma, microdissected, age 53, HPV 16	Cervical cancer_CaCx28	202.4
GSM247883	Cervical cancer, squamous cell carcinoma, microdissected, age 82, HPV 16	Cervical cancer_CaCx54	376.8
GSM247884	Cervical cancer, squamous cell carcinoma, microdissected, age 47, HPV 16	Cervical cancer_CaCx60	183.2
